# Advancing Organ-on-a-Chip Systems: The Role of Scaffold Materials and Coatings in Engineering Cell Microenvironment

**DOI:** 10.3390/polym17091263

**Published:** 2025-05-06

**Authors:** Guido Andrés Ramírez-González, Chiara Consumi-Tubito, Ernesto Vargas-Méndez, Carolina Centeno-Cerdas

**Affiliations:** 1 Master’s Program in Medical Device Engineering, School of Materials Science and Engineering, Costa Rica Institute of Technology, Cartago 30109, Costa Rica; guramirez@estudiantec.cr; 2 Biotechnology Research Center, Costa Rica Institute of Technology, Cartago 30109, Costa Rica; chiaraconsumi@estudiantec.cr; 3 Department of Biochemistry, School of Medicine, University of Costa Rica, San Jose 11501-2060, Costa Rica; ernesto.vargasmendez@ucr.ac.cr

**Keywords:** biocompatibility, hydrogel, PDMS, extracellular matrix

## Abstract

For organ-on-a-chip (OoC) engineering, the use of biocompatible coatings and materials is not only recommended but essential. Extracellular matrix (ECM) components are commonly used as coatings due to their effects on cell orientation, protein expression, differentiation, and adhesion. Among the most frequently used coatings are collagen, fibronectin, and Matrigel, according to the specific cell type and intended OoC application. Additionally, materials such as polydimethylsiloxane (PDMS), thermoplastics, chitosan, and alginate serve as scaffolding components due to their biomechanical properties and biocompatibility. Here, we discuss some of the most employed coating techniques, including SAMs, dip coating, spin coating, microcontact printing, and 3D bioprinting, each offering advantages and drawbacks. Current challenges comprise enhancing biocompatibility, exploring novel materials, and improving scalability and reproducibility.

## 1. Introduction

On-chip systems integrate microtechnology, biomaterials, and cell biology to develop in vitro platforms that aim to mimic the complex interactions occurring in human tissues and living organisms [1,2,3,4]. These microfluidic devices allow for regulation of parameters, such as concentration gradients, cell patterning, mechanical stimulation, and tissue–organ interactions [1].

Organ-on-chip (OoC) and similar systems are typically constructed using materials such as polydimethylsiloxane (PDMS), glass, and other polymers due to their flexibility, transparency, and ease of fabrication [1,5,6]. Despite their widespread use, these materials often exhibit limitations in terms of biocompatibility, and their ability to promote cell adhesion and mimic the physiological microenvironment of living cells accurately [7,8]. Improving these materials is essential to enhance their compatibility with biological tissues, reduce unintended interactions, and create more accurate models of cellular environments. Such advancements could lead to more reliable simulations of human physiology and better predictive tools for drug discovery and biomedical research.

The current goals of the biomedical industry include the involvement in the search and development of accessible biocompatible materials, particularly those with tunable biodegradability and resorption [2,9,10]. To evaluate the effectiveness and safety of products made of such materials, preclinical and clinical evaluations must be carried out.

Preclinical tests using 2D cell cultures and animal modeling present plenty of limitations and concerns to ensure the safety and effectiveness of a drug, medical device, or tissue-engineered construct, such as an adequate simulation due to physiological interactions among tissues and organs, ethical issues, and replicability of results [2,11,12,13].

Moreover, integrating controlled substance-releasing materials into “on-chip” systems presents a promising avenue for advancing biomedical research and therapeutic applications [14,15]. Such materials could enable precise delivery of drugs or signaling molecules, contributing to more closely mimicking the dynamic physiological conditions of human tissues, and improved simulation of cellular responses, enhanced drug testing accuracy, and potential applications in personalized medicine. However, challenges, such as ensuring material biocompatibility, as well as maintaining or regulating release kinetics and preventing unintended interactions with the microenvironment, must be addressed [16]. Furthermore, the complexity of designing systems that can reliably replicate the intricate interplay of biological processes poses significant technical hurdles. Overcoming these obstacles would require interdisciplinary collaboration and innovative approaches in material science and bioengineering.

In recent years, several OoC platforms have been developed, mostly for the heart [11], liver [17,18,19], gut [17,20,21,22], and kidney [23]. Despite significant progress in OoC technology, challenges remain in standardizing optimized fabrication methods, ensuring reproducibility, and replicating the dynamic interactions present in human tissues [24]. While PDMS remains a widely used material due to its already mentioned optical transparency and biocompatibility, alternative materials, such as thermoplastics, extracellular matrix (ECM)-mimicking polymers, and biofunctional coatings, are being explored to improve biomimicry and physiological relevance [6,25,26,27]. Additionally, integrating microfabrication techniques with advanced imaging and analytical tools is expected to enhance the precision of multi-organ models, supporting applications in personalized medicine and drug discovery [11].

This review examines the latest advancements in scaffold materials and coatings for OoC systems, highlighting their role in engineering the cellular microenvironment and their potential to improve the physiological relevance of in vitro models. By addressing current limitations and exploring novel biomaterial approaches, OoC technology can further bridge the gap between preclinical testing and clinical applications, ultimately improving the predictive accuracy of biomedical research.

## 2. Organ-on-a-Chip Device Materials

The material properties play a pivotal role in the construction and functionality of organ-on-chip systems. Some synthetic polymers, for example, offer advantages, such as optical transparency, gas permeability, and ease of microfabrication, making them a popular choice for creating microfluidic channels; however, they also present limitations. Understanding and optimizing these material properties are essential to improve the physiological relevance and reliability of organ-on-chip systems.

### 2.1. PDMS

PDMS, a silicone-based polymer, plays a pivotal role in the field of OoC technology. It is widely used due to its desirable properties. First, its biocompatibility makes it non-toxic and suitable for 3D cell culture [5,20,23,28,29], as seen in different OoC systems. Second, its transparency allows real-time observation of cell behavior [30]. Third, PDMS is elastomeric, enabling precise molding into intricate shapes [31,32,33]. Fourth, it exhibits gas permeability, allowing oxygen and carbon dioxide diffusion [5]. Finally, its ease of fabrication via soft lithography techniques enables customizable microfluidic designs [34,35].

In OoC technology, researchers commonly use PDMS-based chips, which comprise microchannels, chambers, and semipermeable membranes, for cell culture, fluid shear stress studies, chemical gradients, and multi-organ systems, while addressing challenges such as hydrophobicity management, long-term stability, and its tendency to absorb small hydrophobic molecules, which can affect experimental outcomes [36]. As they refine designs and delve into bioprinting, PDMS continues to propel the advancement of OoC technology [5].

### 2.2. Polycarbonate

Polycarbonate is widely used in biomedical applications due to its biocompatibility and mechanical properties. Its chemical structure consists of bisphenol-A molecules linked by carbonate groups, and it is commonly employed in the production of food and drug packaging components, as well as medical devices [37].

In OoC technology, polycarbonate has been used in the development of membranes, including chips designed to mimic fetal membranes at the mother–child interface [38]. Additionally, its gas impermeability makes it suitable for models involving small molecule treatment studies [39].

Using polycarbonate as a gut-on-a-chip material allowed Caco2 cells to form tight junctions. This was proven to be a good model to study the anaerobic bacterium *Blautia coccoides*, showing that the gas-impermeable properties attributed to polycarbonate effectively prevent oxygen infiltration during chip fabrication and cell culturing [39].

Furthermore, polycarbonate is ideal for DNA heat-cycling applications due to its transparency to visible light and its high glass transition temperature (approximately 145 °C). Its potential for biological applications was further evidenced by a study on RPTEC/HUVEC cell co-culture, where cell viability was significantly higher in polycarbonate chips compared to transwell systems [40,41].

### 2.3. Polyurethane

Polyurethane is widely used in modern OoC systems due to its biocompatibility, high tensile strength, durability, toughness, resistance to degradation, and flexibility [42,43]. Its flexibility makes it a preferred choice over rigid thermoplastics for systems that replicate soft tissues requiring dynamic strain and stretchability, such as the lungs [44] and heart [45].

To mimic cardiac tissue, researchers have used polyurethane to create nanofibers that orient cells in alignment resembling muscle tissue [46]. A study by Kobuszewska et al. (2021) demonstrated that cell alignment closely followed the nanofiber arrangement, with a maximum deviation of 30° [47].

Another study, by Mitta et al. (2024) [44], replicated lung tissue stiffness using polyurethane membranes to model alveolar epithelial cells. These membranes supported cell proliferation and increased SFTPA1 expression compared to culturing cells on PET, indicating enhanced surfactant production and a closer resemblance to native alveolar cells. Additionally, the membranes withstood breathing-related stresses from both human and mouse models [44].

### 2.4. Poly-Methyl-Methacrylate (PMMA)

PMMA is a polymer widely used in cell culture, showing excellent compatibility without requiring coatings or surface treatments. This avoids the formation of cytotoxic products that can occur when thermoplastics are exposed to UV light or ozone [48]. Due to its mechanical and optical properties, PMMA serves as an ideal substrate for OoC system fabrication [49]. Another notable advantage of PMMA is its behavior during laser cutting, where it vaporizes into gaseous components. This process ensures clean cuts without burnt edges, preserving optical quality and cell viability [49].

In a study by Schneider et al. (2021) [50], PMMA was used to create a circular peristaltic on-chip pump. Stacked discs formed a channel where HUVEC cells were seeded, demonstrating good viability and CD31 expression. PMMA’s rigidity contributed to the stability and durability of the pump system [50]. However, since PMMA is impermeable to gases, OoC system channels made with this material should be short to allow adequate oxygen diffusion through the inlet and outlet [48].

### 2.5. Cellulose

Cellulose-based scaffolds for tissue engineering have gained attention in recent years due to their high-water retention, biocompatibility, and natural origin, offering environmentally friendly alternatives to petroleum-derived polymers [51,52].

In OoC systems, cellulose has shown promising results as a surface for cell growth, despite its limited flexibility, durability, and strength [53]. To address these limitations, cellulose membranes are often combined with other materials, such as PDMS, acetate, and PMMA [53,54].

Li et al. (2022) [54] used cellulose alongside PMMA in 3D-printed scaffolds to evaluate the viability of multiple cell types in organ-like structures. After seven days in culture, HepG2, HUVECs, AS49, and HK-2 cells demonstrated survival rates above 90% and expressed proteins related to their specific functions, as confirmed by immunofluorescence analysis [54].

Moreover, Hospodiuk-Karwowsk et al. (2022) [55] developed a pancreas-on-a-chip model using carboxymethyl bacterial cellulose to assess cell viability, insulin production, and the expression of pancreatic markers. Cells cultured with cellulose-containing hydrogels exhibited the highest insulin production, up to four times greater than other treatments [55].

### 2.6. Chitosan

Chitosan is a polysaccharide obtained from the complete or partial N-deacetylation of chitin, and it is particularly suitable for creating membranes and scaffolds under conditions that are not overly harsh and noncytotoxic, making it an ideal choice for OoC systems [56,57,58].

For instance, Tibbe et al. (2018) [56] developed a microfluidic chip, including a temporary chitosan-based membrane to separate astrocytes from endothelial cells but allowing direct cell–cell contact between the cell types [56]. Since chitosan is a polysaccharide that forms a gel-like solid upon deprotonation, by creating an interface between a basic buffer solution and an acidic chitosan solution, the chitosan gets deprotonated at the interface, forming the membrane, which acts as a physical barrier for cell culture and can be removed afterwards by flushing an acidic solution. The researchers added an immunofluorescent cell tracker to follow astrocyte position and morphology, and none of them were influenced by the removal of the membrane. According to the researchers, this possibility of membrane fabrication and removal can be used to create membrane-free co-cultures in a microfluidic device for OoC research.

Chitosan has also shown good results when used in a composite with alginate [58,59,60]. Upadhyay et al. (2024) [58] used a combination of chitosan and alginate biopolymers and decellularized ECM (dECM) as a bioink additive in the development of scalable OoC using a microfluidic platform. The bioink was tested with native chondrocytes and mesenchymal stem-cell-induced chondrocytes using biopolymers of alginate and chitosan composite hydrogels [58]. They used 2D and 3D biomimetic tissue construction approaches to characterize several aspects of cells and found that the bioink significantly increased both phenotypic and genotypic expression, with a statistical significance level of *p* < 0.05.

In another study by Chiu et al. (2012), a chitosan–alginate composite was used to make a micropattern of arrays on a chip for myocardium modeling [57]. The results showed that cardiomyocytes, cultured on gels featuring 10 μm-wide grooves, successfully formed a well-structured 3D beating tissue concerning those in the smooth surface [49]. Given the perpendicular orientation of the grooves to the electrodes, the cells were able to align with the electric field lines, thereby experiencing the maximum voltage differential.

### 2.7. Alginate

Alginates are polysaccharides composed of β-d-mannuronic acid (M) and α-l-guluronic acid (G), organized in homogenous blocks (-GGG- and -MMM-) or alternating blocks (-MGMG-), with a G content ranging from 30% to 70%, depending on the species and part of the marine algae of origin [61]. Their biocompatibility, non-toxicity, hydrophilicity, ease of blending, and low immunogenicity make them widely used biomaterials in tissue engineering, with applications such as wound healing, bone graft substitutes, cell therapy, and ventricular support in dilated cardiomyopathy [61,62,63].

Pangjantuk et al. (2024) reported that three-dimensional hydrogels of alginate and hyaluronic acid (AL-HA) promote cell proliferation, enabling the cultivation of cells in spheroids within the hydrogel matrix [64]. The results showed a high cell survival rate, reaching 77.36% over a 14-day cultivation period. This 3D culture approach proved effective in maintaining the viability and functionality of human mesenchymal stem cells, highlighting its potential for biomedical applications.

Once crosslinked with Ca^2+^, sodium alginate hydrogel exhibits high mechanical strength and convenient elasticity, making it an ideal support material for 3D cell cultures in vitro. However, to better mimic the ECM and enhance its biocompatibility, it needs to be combined with other natural polymers [65].

The incorporation of agarose, gelatin, and fibrinogen into alginate hydrogels can reduce the pore size of the matrix, providing a greater number of binding sites for cells, resulting in increased cell survival and proliferation capacity [65]. However, moderate addition is crucial, as excessive amounts may overly reduce pore size, limiting cell proliferation and migration, as well as diffusion of nutrients and waste metabolites. Optimized alginate-based composite hydrogels with an appropriate microstructure enhance cell survival and increase cell density, reduce culture costs, and create a favorable environment for cellular interactions, ultimately promoting the formation of tissues or organs [65,66,67].

## 3. Anchorage Dependence as a Property and the Role of the Substrate

Anchorage dependence is a property of cells implying their requirement to be attached to a surface to survive and proliferate and is also a critical factor in determining the shape, behavior, and fate of cells.

Cells of most animal tissues are attached together and to the extracellular matrix (ECM) [68]. The ECM is a dynamic scaffolding system composed of multiple macromolecules organized in a tissue-specific manner that contributes to the microenvironment and homeostasis of the cells. The components of the ECM integrate in a structurally stable composite, contributing to the mechanical properties of the tissue, which is likewise required for tissue regeneration and other biological events [69,70,71].

It has been shown that the spatial distribution of the ECM helps guide the orientation of the cell division axis, and it also plays an important role in mechanical stimuli, thanks to the activation of mechanical sensors like integrins [69,70,72].

The composition of the ECM varies according to the type of cells, tissues, and organs, but it is usually composed of complex proteins and carbohydrates, including collagens, fibronectin, laminin, and hyaluronic acid proteoglycans and glycosaminoglycans, among others [68,73]. The interaction between collagen and the cellular scaffold is essential for maintaining tissue integrity and promoting cell survival. Additionally, collagens influence cell behavior by transmitting biochemical signals that regulate cell proliferation, differentiation, endurance, and continuity [74].

In bioengineering, the ECM is exploited in several ways, mostly as scaffolds and coatings, e.g., to improve cell adhesion, to promote tissue regeneration in medical applications, and to create in vitro models for physiological study, preclinical testing, and personalized medicine [17,74,75,76,77,78].

## 4. Hydrogels as Biocompatibility-Enhancing Coatings

The properties of biocompatibility and biodegradability displayed by the components of the ECM, including hydrogels like the above-mentioned collagen, fibronectin, hyaluronic acid, and others, are currently exploited in a variety of applications. Hydrogels are pivotal in enhancing OoC applications due to their capacity of improving cell adhesion, proliferation, migration, and overall biocompatibility. These water-swollen polymers provide a supportive, hydrated environment that facilitates nutrient and waste exchange, critical for cell viability and function [17,26,74,75,76,77,78]. By tailoring the biochemical and physical properties of hydrogels, researchers can create specific microenvironments that promote the desired cellular behaviors, such as enhanced adhesion and proliferation [79]. Additionally, hydrogels can be engineered to include bioactive molecules, such as growth factors, which further support cell differentiation and physiology, ultimately leading to tissue formation.

Coating substrates with hydrogels can be achieved through various techniques, such as dip coating, spraying, and spin coating, allowing for the utilization of their unique properties, including lubricity, biocompatibility, and antifouling resistance. These characteristics, combined with the specific mechanical properties of the substrate, such as stiffness, toughness, and strength, provide great versatility and advantages in multiple applications [80]. In particular, the application of hydrogels in OoC systems is highly beneficial, as it enables the replication of complex, dynamic interactions, similar to those of living tissues, which is essential for accurately modeling physiological and pathological processes [81]. Additionally, recent studies have demonstrated that biocompatible hydrogel coatings can significantly enhance the antifouling and lubricious properties of materials commonly used in medical and biomedical devices, further supporting their potential for improving OoC materials [82]. 

## 5. Molecules Used to Resemble ECM

### 5.1. Collagen

Collagen (I to XXVIII) is a structural protein present throughout the human body, conformed by three polypeptide fibrils that form a triple helix structure, with a diameter of 10–500 nm each and a molecular weight around 285 kDa [40]. Even though there are 28 collagen types identified today, the most abundant in the human body are collagen I, II, and III [40,83,84].

In bioengineering, collagen has been widely used to mimic the ECM in scaffolds for tissue regeneration [25] and organs-on-a-chip [20,21,85] due to its mechanical strength and cellular compatibility, resembling the natural environment of tissues [2]. Epithelial cells are typically seeded and cultured on collagen coatings, in organ-on-a-chip (OoC) models, as well as in implants, tissue engineering constructs, and other scaffolds [86,87,88]. This is due to the ubiquity and abundance of collagen in human tissues, making it an ideal substrate for cell attachment and proliferation.

Jeon et al. (2020) used collagen I alongside Poly-D-Lysine in a microfluidic chip to improve cell and collagen adhesion; then, they compared the differentiation grade under static and flow conditions and looked for the establishment of a collagen-rich basal membrane separating the cell types successfully [21]. The results showed that the combination of three factors (the presence of collagen, co-culture with endothelial cells, and maintenance under flow conditions) led to a high differentiation rate and a strong barrier of CaCO_2_ epithelial cells. More specifically, the latter led to an increase in the length of the villi expressed by the cells, with a peak of over 35 μm in size and an impedance of 59 Ω·cm^2^.

In another study, collagen membranes were made for OoC engineering using ARPE-19 epithelial cells and human pluripotent stem-cell-derived endothelial cells (hiPSC-ECs) and showed expression of adhesion markers (VE-cadherin for hiPSC-ECs and ZO-1 for ARPE-19) alongside the formation of monolayers [83].

Additionally, collagen has been shown to improve stability on cell viability when used as polymers for epithelial cells, such as CaCO_2_, and to increase the expression of epithelial barrier markers [89]. In a study by Wang et al. (2018), they compared the viability of cells seeded in chips without collagen with transwell plates and with collagen membranes, and despite that no significant changes were seen between the three treatments regarding viability, collagen showed the most stable results [89]. In the same study, they compared tight junction protein expression, F-actin, and ezrin in cells using ZO-1, phalloidin, and ezrin staining, and for all markers, the fluorescence intensity was significantly higher when using collagen. Epithelial cells are the ones that are seeded and reproduced in collagen coatings, due to the abundance of said protein in these kinds of tissues in humans.

### 5.2. Matrigel

Matrigel, extracted from the Engelbreth–Holm–Swarm mouse sarcoma, is a gelatinous protein mixture used in cell culture, mainly composed of laminin, collagen IV, entactin, and heparin sulfate proteoglycan perlecan [30]. Despite not being used in a clinical way due to its tumoral origin, it is widely used in in vitro models and has shown great success in 3D culturing [90]. This coating is especially, but not restricted to, being used in tumor cell research, because it provides a supportive microenvironment that facilitates the growth, differentiation, and three-dimensional organization of tumor cells. This enables the modeling of invasive behaviors characteristic of cancer cells. For instance, Passaniti et al. (2022) [91] compiled evidence on how co-injecting tumor cells with Matrigel enhances tumor take and growth, allowing for the development of animal models that closely mimic human cancers [91].

Furthermore, Matrigel supports the formation of tumor spheroids, which are three-dimensional aggregates of tumor cells that replicate the architecture of solid tumors in vivo. The addition of Matrigel improves the growth environment of these spheroids, making them more representative of in vivo tumor conditions [92].

Examples of devices coated with Matrigel include items for a heart-on-chip, where this substance was used with hiPSC-CMs because of showing the best proliferative activity and viability, by exhibiting 94.3 ± 0.3% and 98.2 ± 0.2% living/dead ratios under static and dynamic conditions, respectively [28].

Matrigel offers biocompatibility, mimics native tissues, and finds applications in OoC technology, including microfluidic devices, drug delivery research, toxicology studies, and multi-organ systems [5,93]. Researchers appreciate its natural composition and ability to replicate under in vivo conditions.

In a study by Carvalho et al. (2019), Matrigel contributed to the endothelial invasion thanks to its vascular endothelial growth factor (VEGF), for which the cells seemed to follow a chemotactic signal toward the Matrigel source even though cell migration from the endothelium is not physically constrained in any direction by their system design [94]. Moreover, Dolega et al. (2015) showed Matrigel to promote clusters of RWPE1 prostate cancer cells within 6 to 8 days [95].

### 5.3. Fibrin and Blood Plasma

Fibrin is a biopolymer formed from fibrinogen when stimulated with thrombin, which cleaves fibrinopeptides from fibrinogen, producing fibrin fibers that assemble in a crosslinked mesh [96]. This mechanism proceeds by exposing a polymerization site with a binding pocket to form an association, causing double-stranded twisting fibrils [97]. Various crosslinks form, conferring mechanical and elastic properties, giving fibrin structural integrity, and stabilizing the clot [96,97]. When in soluble form as fibrinogen, it is part of the blood plasma alongside other components, such as thrombin, fibronectin, fibrinolysis inhibitors, molecules of cell adhesion [98], as well as platelets and growth factors.

Both fibrin and blood plasma have shown promising results for tissue regeneration and cell proliferation [97,99]. In a study by Yang et al. (2020) [97], cell viability was assessed using an MTT assay (3-[4,5-dimethylthiazol-2-yl]-2,5-diphenyltetrazolium bromide), which is a colorimetric test that measures cell viability and is also used to study cell proliferation and cytotoxicity. Stem cells were cultured on polycaprolactone (PCL)/fibrin scaffolds, in proportions varying from 0:100 to 30:70, respectively, finding that fibrin could effectively reduce the cytotoxicity of PCL [97]. In another study, rat cardiomyocytes were seeded in a PDMS chip with a fibrin/collagen hydrogel with a proportion of 85:10, in which this, alongside a specific array design of posts in the scaffold, allowed the expression of connexin 43, correct alignment of sarcomeres, optimal width and alignment of human stem-cell-derived cardiac fibers, and spontaneous contractile behavior [99].

In a study regarding blood plasma scaffolds, when put together with collagen, adipocyte stem cells showed proliferative activity, increasing by 1.23 times their number after 72 h of culture [97].

### 5.4. Fibronectin

Fibronectin is a glycoprotein with a size of 230 to 270 kDa consisting of monomers classified as type I, II, or III [100]. This glycoprotein can be produced either by hepatocytes, in which case it is called plasma fibronectin, or by fibroblasts, chondrocytes, myocytes, and synovial cells, where it is known as cellular fibronectin [100,101].

This molecule plays an important role in cell adhesion and migration, transmitting also mechanical and biochemical stimuli for cell morphogenesis, as it is part of the extracellular matrix, mainly on endothelial cells [102,103]. Because of this, fibronectin has been used as a coating for organ-on-a-chip engineering [19,23,104] and cell culture [105,106].

In a study by Bas Cristóbal-Menéndez et al. (2022) [23], fibronectin coating in channels where HUVECs were seeded alongside a kidney organoid increased the total area of presence of the melanoma cell adhesion module (MCAM+) and platelet endothelial cell adhesion molecule (PECAM+) immunofluorescence staining and their mutual colocalization compared to what was seen in a transwell plate [23]. Both markers are used as signals of endothelization, related directly to the main goal of the study, which was promoting vascularization on the kidney organoids. This result may not be directly related to the coating, but mostly to the contact between the organoid and the HUVEC cells. Nevertheless, fibronectin coating has been shown to improve adhesion of integrin-expressing cells [107,108], such as HUVECs [108].

Fibronectin was also used for a liver-on-a-chip platform, in which Xie et al. (2021) showed viability above 80% for both HepG2 cells and HUVECs [104]. Also, in the same study, HUVECs’ nuclei showed a homogeneous distribution while having a high expression of CD31 and VE-cadherin, which indicates the formation of a functional endothelial barrier [14,16,44,45,59].

### 5.5. Decellularized Extracellular Matrix

In an OoC system, decellularized ECM (dECM) is frequently paired with primary or stem cells to simulate organ-specific functions [109]. This approach allows researchers to develop models that more accurately mimic human physiological responses to drugs, materials, diseases, or other variables, surpassing the predictive capabilities of conventional 2D cell culture systems [110,111].

This coating is used to produce bioinks that recreate tissue-specific microenvironments. Combined with 3D-printing technology, they enable the precise and reproducible fabrication of platforms [112]. Bioinks involve formulations that blend decellularized matrix materials with biocompatible substances, creating a medium that supports cell growth while mimicking the native tissue environment [113,114,115]. These bioinks allow 3D printing of complex, highly accurate structures, such as vascular networks or the layered organization of organs [116].

**Figure 1:**
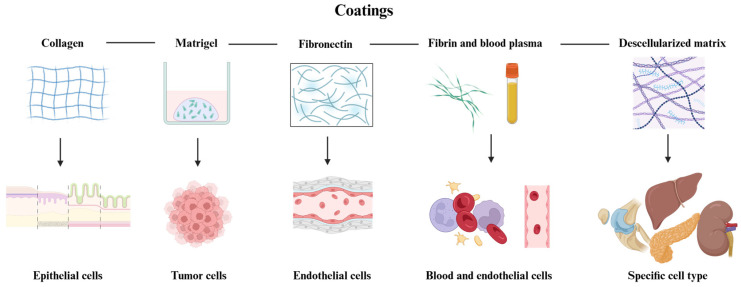
**Representation of the coating hydrogels reviewed in this paper**. As shown, collagen is usually used for experiments with epithelia, Matrigel for tumors (due to its cancerous origin), fibronectin is used for endothelium and vascularity experiments, while fibrin and blood plasma are commonly used in studies involving blood cells and endothelium. Decellularized ECM is used to support the growth of several cell types, depending on the tissue of origin. Created in BioRender. Ramirez, G. (2025): https://BioRender.com/r96y578.

The utility of bioprinting and the specificity and biological influence of the ECM are evident in studies like the one of Kim et al. When comparing dECM and collagen as coatings for a PDMS chip to develop a multi-OoC system to study type 2 diabetes (T2D), their dECM successfully replicated T2D pathological conditions, downregulating pIRS-1, pAMPK, and GLUT-4 in adipose tissue, and GLUT-2 in the pancreas and liver, mirroring impaired glucose signaling seen in diabetes. In contrast, collagen increased IRS-1 and AMPK phosphorylation and enhanced GLUT-2 and GLUT-4 expression, indicating a response to glucose availability rather than the metabolic dysfunction typical of diabetes [112]. (Check Figure 1 and Table 1 for more details on hydrogels used as coatings).

## 6. Addition of Coatings on Chips

Although there are many techniques for applying coatings to biomaterials, for OoC purposes some are more commonly used than others due, for example, to the way the chips are prepared or assembled. Nevertheless, OoC represents a promising alternative to evaluate bioactive surface coatings based on stimuli-triggerable approaches, the incorporation of bioactive compounds in drug-releasing strategies, or to provide corrosive resistivity, anti-inflammatory, and antimicrobial properties to medical devices.

OoC often relies heavily on the coating using hydrogels to ensure its success, simply to allow cell attachment or even to enhance cellular behaviors and improve the biomimicry of the device [93]. The selection and application of coatings are crucial for creating a supportive environment for cells and ensuring the functionality of the OoC (see Table 2 for the main aspects of common techniques). The coating procedure must also consider the sterilization method and the stability of the molecules used.

### 6.1. Self-Assembled Monolayers (SAMs)

Self-assembled monolayers (SAMs) are highly organized layers formed by the adsorption of molecules onto a substrate [118]. This technique allows precise control over the chemical functionality of the surface, making it possible to tailor the surface properties to promote specific cell behaviors [119]. SAMs are typically used for their ease of application and the ability to create uniform, stable coatings that can be modified to include bioactive molecules [118,120].

Maoz et al. (2017) [121] used SAMs for the creation of the transepithelial electrical resistance with multielectrode array chips, allowing for the optimization of interactions between materials and cultured cells. SAMs were formed using APTES (3-aminopropyltriethoxysilane) and GLYMO (3-glycidoxypropyltrimethoxysilane). The process begins with an oxygen plasma treatment, promoting the activation of the surfaces of the materials used in the device, including PDMS, polyethylene terephthalate, polycarbonate, and silicon nitride [121]. According to the researchers, this step generates reactive functional groups on the surfaces, facilitating the chemical adhesion of the coating, forming stable and uniform monolayers.

SAMs derived from APTES provided reactive amino groups (-NH_2_), while those generated with GLYMO introduced epoxy groups on the surface, enhancing the adhesion of biomolecules and the formation of covalent bonds between the functionalized layers [122].

### 6.2. Layer-by-Layer (LbL) Assembly

The layer-by-layer (LbL) assembly technique involves the sequential adsorption of oppositely charged polyelectrolytes onto a substrate [123]. This method enables the fabrication of multilayered coatings with nanoscale precision with the possibility of incorporating a variety of materials, including proteins, peptides, and nanoparticles [124,125]. This technique is particularly useful for creating complex, multifunctional surfaces that can mimic the ECM [126].

Aor et al. (2020) made a chip consisting of multiple layers of chitosan and hyaluronic acid over polycarbonate, on top of which HUVECs were seeded [127]. These cells were able to initiate tubulogenesis thanks to the micropatterned platform.

### 6.3. Spin Coating

For this technique, a solution of the coating material is deposited onto a substrate, which is then spun at high speed to spread the material uniformly. This method is commonly used for applying thin polymer films of ECM proteins or synthetic polymers that support cell attachment and growth and is valued for its simplicity and ability to produce highly uniform coatings [128,129,130,131].

Quirós-Solano (2018) et al. spin-coated transferable porous PDMS membranes to produce OoCs [29]. This approach aimed to overcome the limitations associated with replica molding, expanding the applicability of most OoCs while improving their scalability and reproducibility. The membranes proved to be biocompatible and suitable for cell culture, as demonstrated by the successful growth of HUVECs and MDA-MB-231 (MDA) cells.

### 6.4. Dip Coating

Dip coating involves immersing the substrate into a solution of the coating material and then withdrawing it at a controlled speed, allowing a thin film to form as the solvent evaporates [132]. This technique is advantageous for its ability to coat substrates with complex geometries and its suitability for large-scale production [133]. The dip coating is often employed for the deposition of hydrogels and other biocompatible materials that provide a supportive matrix for cells [134].

For example, to protect sugar fibers from early dissolution during cell seeding and the hydrogel casting process, Nie et al. (2024) made a hydrophobic protective coating using this technique, which was convenient because of the complexity on the geometry of the scaffolds [135].

### 6.5. Microcontact Printing

Microcontact printing uses a patterned stamp to transfer molecules onto a substrate [113,136,137]. This method allows precise spatial control over the deposition of the coating material, making it possible to create micropatterned surfaces that guide cell organization and function, making this technique particularly useful for applications that require the creation of specific cellular microenvironments within the OoC.

Sun et al. (2024) made (in just 8 min) a geometric pattern in a PDMS ink spin-coated film and then tested its efficacy as a OoC platform with MCF-7 breast adenocarcinoma cells [136]. The results showed that these cells restricted their growth to the designated areas of the structure in a 3D-specific arrangement, as is usually seen in tumors. This presents microcontact patterning as an efficient technique for mass production of micro-arrangements required for OoC platforms.

### 6.6. 3D Bioprinting

Three-dimensional bioprinting enables the simultaneous deposition of biocompatible materials and living cells, incorporating physiologically relevant features, such as precise cellular arrangements, to replicate cellular diversity and microstructure with consistency [138]. Common techniques include extrusion-based bioprinting, micro-extrusion, inkjet bioprinting, stereolithography (SLA), and laser-induced forward transfer (LIFT) [74,113,139,140].

Extrusion-based bioprinting is particularly effective for constructing cell-laden structures in tissue engineering, ensuring fabrication precision and cell viability [139]. This method involves the layer-by-layer deposition of bioink filaments extruded continuously through a nozzle [141]. These systems typically include a material reservoir, print head, movable printing platform, and a positioning mechanism for precise movement along the x, y, and z axes [139].

Inkjet bioprinting, on the other hand, employs nozzles activated by physical stimuli to eject tiny droplets of bioink onto a substrate, forming intricate 3D tissue structures. This technique excels in high-resolution printing and precise cell placement [142,143].

The printability of biomaterials depends on their mechanical properties, including rheological behavior, bioactive components, and degradation characteristics [138]. For instance, Abudupataer et al. (2019) [144] demonstrated the use of bioprinters to deposit a bioink containing endothelial cells, smooth muscle cells, and gelatin methacryloyl in a microfluidic chip. The printed cells showed high viability and increased vascular protein expression compared to traditional 2D cell culture methods [144]. Furthermore, Upadhyay et al. (2024) demonstrated that bioink loaded with cell-containing biopolymers significantly enhanced phenotypic and genotypic expression, optimizing conditions for hyaline cartilage development in culture [58]. This advancement facilitated the study of mechanosensitive properties in microfluidic cartilage-on-a-chip systems.

## 7. Comparative Analysis of Coating Techniques

**Figure 2:**
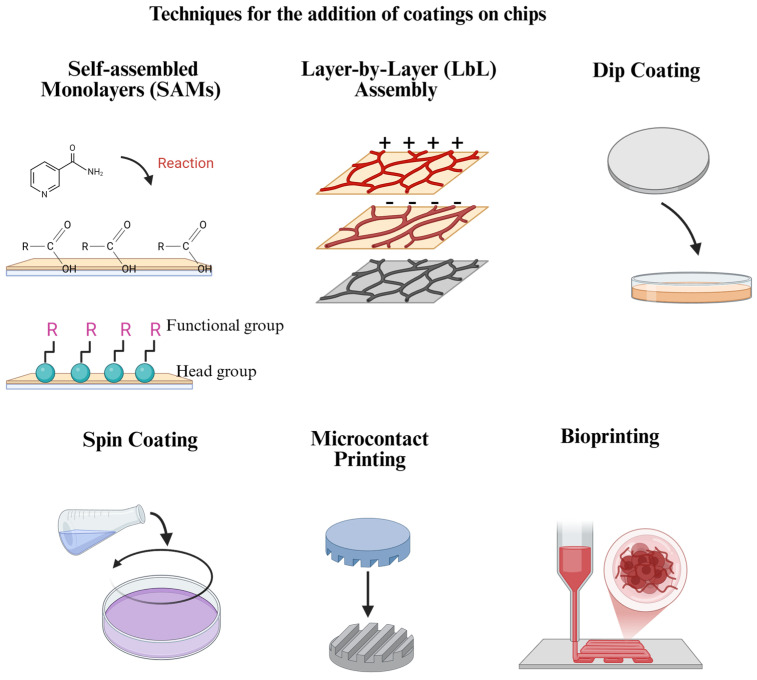
**Frequently used coating addition techniques for OoC platforms**. Each technique has advantages and limitations in terms of costs, coating stability, reproducibility, and other aspects, which must be considered when selecting the one to use. Created in BioRender. Ramirez, G. (2025): https://BioRender.com/q32n598.

The choice of coating technique depends on several factors, including the desired properties of the coating, the type of substance, the sterilization method and cells being used, as well as the specific application of the OoC. Each technique offers unique advantages and limitations (Check Table 2 and Figure 2 for further details).

**Table 2:** Techniques used for coating addition on chips.

Technique	Advantages	Limitations	References
Self-assembled monolayers (SAMs)	Provides precise control over surface chemistry.	Limited possibility to create thick coatings.	[118]
Layer-by-layer (LbL) assembly	Offers versatility and the ability to create complex, multifunctional surfaces combining several polymers.	Can be time-consuming and requires multiple steps.	[122,127]
Spin coating	Produces uniform thin films.	May not be suitable for substrates with complex geometries.	[11,128]
Dip coating	Suitable for complex geometries and large-scale production.	May result in less uniform coatings, which can, in turn, affect the results and reproducibility.	[122,132]
Microcontact printing	Allows for precise patterning.	Limited by the resolution of the stamp and the complexity of the patterns that can be achieved.	[136]
3D Bioprinting	3D bioprinting enables the precise arrangement of cells and biomaterials, replicating native tissue structures with high fidelity.	Requires bioinks with appropriate mechanical, rheological, and degradation properties.	[144]

## 8. Challenges and Future

The main difficulties of developing an OoC platform are mostly related to the simulation of a physiological microenvironment while ensuring system functionality, reproducibility, and cost-effectiveness [145,146]. An example related to the functionality and reliability of materials used for OoC platforms is PDMS, as this material tends to absorb small hydrophobic molecules, which can interfere with drug testing, and the cells within the chip may not accurately display the same response as seen in vivo [147].

One of the primary challenges is biocompatibility, as materials must be noncytotoxic and with a low to non-immunogenic response [148,149]. Beyond compatibility, materials need to replicate the mechanical, chemical, and physical properties of native tissues, such as elasticity, porosity, and surface characteristics [150,151]. Hydrogels are promising for mimicking soft tissues but often lack the mechanical stability required for long-term applications [151,152].

The integration of sensors into OoC systems poses additional complexity, as materials must accommodate electrodes or optical tools for real-time monitoring without introducing artifacts or compromising material properties [153].

One specific challenge in coatings is achieving reproducibility and scalability in the distribution and thickness of the coating layer. In large-scale manufacturing, ensuring that each chip has a uniform coating thickness can be time-consuming and resource-intensive. Variations in coating thickness can lead to inconsistencies in product functionality, potentially resulting in higher rejection rates and increased manufacturing costs and losses.

New research regarding OoC is leading to the search for new materials and methods of fabrication that ensure a more precise simulation of the desired organ, as well as better reproducibility while reducing the costs of manufacturing [154,155,156]. One example is 3D bioprinting, such as the technique used by Conceição et al. (2022) [157], which was an innovative three-dimensional, printing-based, multi-compartment microfluidic platform that allowed both selective and dynamic multicellular paracrine signaling between sympathetic neurons, bone tropic breast cancer cells, and osteoclasts cultured together to make a metastatic bone cancer model [157].

## Figures and Tables

**Table 1:** Evaluation of some cell scaffold and coating materials, and their influence on cell behavior.

Compound	Application	Evaluated Aspects	Main Findings	Additional Comments	References
Collagen Type I	Membrane for organ-on-a-chip engineering.	Protein expression on the epithelial barrier.	Expression of adhesion markers and formation of monolayers of cells.	Rat tail collagen type I was used for this experiment alongside ARPE-19 and hiPSC-EC cells.	[83]
	Gut-on-a-chip for inflammation studies.	Differentiation with the expression of markers, size of villi, and impedance.	Improvements in differentiation, formation of epithelial barrier, and villi size while in co-culture with HUVECs and constant flow rate. Impedance was 59 Ω·cm^2^.	Poly-D-Lysine was added to improve collagen attachment and cell adhesion. Caco-2 cells were used.	[21]
	Membrane for organ-on-a-chip viability.	Cell viability.	Cell viability was more stable with the use of collagen. Expression of markers such as F-actin and ZO-1 was higher on collagen membranes.	Caco-2 cells were used in this test.	[89]
Matrigel	Duodenal organoid.	Impedance and expression of differentiation markers.	One-way impedance showed results equivalent to real live duodenal tissue (*p* < 0.05), with an impedance of around 50 Ω·cm^2^. Solute carrier transport expression (SCT) like living tissue.	Duodenal cells were used in this study.	[117]
Fibrin	Cardiac tissue-on-chip.	Modeling of cardiac tissues.	Among several hydrogels used, the one with mainly fibrin was the optimal to enhance cell elongation and tissue formation.	Rat cardiac cells. hESC and hiPSC-derived cardiomyocytes were constantly validated with immunofluorescence.	[99]
	Polycaprolactone/fibrin scaffold.	Expression of markers, cell survival, and proliferation.	High cell viability where the proportion of fibrin was bigger. Immunostaining showed good cell proliferation.	Mesenchymal stem cells. Live/dead cell assay.	[97]
Fibronectin	Kidney-on-a-chip.	Expression of vascularization markers.	Vascularization was better in co-culture with HUVECs and dynamic flow than the transwell plate.	iPSC-derived kidney cells were used for this approach.	[23]
Decellularized extracellular matrix.	T2 diabetes model on a chip.	Expression markers.	Significant increase in T2 diabetes markers compared to cells grown in collagen.	Human adipose-derived stem cells.	[112]
